# Association between depressive symptoms and parental stress among mothers and fathers in early parenthood: A Swedish cohort study

**DOI:** 10.3109/03009734.2016.1143540

**Published:** 2016-03-04

**Authors:** Birgitta Kerstis, Eva Nohlert, John Öhrvik, Margareta Widarsson

**Affiliations:** a School of Health, Care and Social Welfare, Mälardalen University, Västerås, Sweden; b Centre for Clinical Research, Uppsala University Västmanland County Hospital, Västerås, Sweden; c Department of Medicine, Karolinska Institutet, Stockholm, Sweden

**Keywords:** Fathers, gender, mothers, parental role models, sense of coherence

## Abstract

**Aim:**

To determine whether there is an association between depressive symptoms and parental stress among mothers and fathers during early parenthood in Sweden.

**Methods:**

In this study, 401 mothers and 396 fathers (393 couples) were included; the Edinburgh Postnatal Depression Scale and the Sense of Coherence Scale were measured 3 months after childbirth, and the Swedish Parenthood Stress Questionnaire and the Sense of Coherence Scale after 18 months. Complete data for multivariable analysis were available for 264 mothers and 252 fathers.

**Results:**

The mothers estimated greater total depressive symptoms and parental stress than the fathers did. Both the mothers and the fathers had the greatest level of stress in the sub-area ‘Role restriction’. The mothers had the lowest level of stress in the sub-area ‘Social isolation’ and the fathers in the sub-area ‘Incompetence’. The mothers perceived greater levels of stress than the fathers did in all sub-areas except for ‘Social isolation’, where the fathers perceived higher stress. There was an association between the parents’ depressive symptoms and parental stress. The parents’ own depressive symptoms at 3 months and sense of coherence and the partners’ parental stress at 18 months were positively associated with the parental stress at 18 months in univariable and multivariable analyses.

**Conclusions:**

Understanding the relationship between depressive symptoms and parental stress is important for health professionals so they can offer parents adequate support in early parenthood to optimize the conditions for raising a child. This knowledge should also be communicated to the parents.

## Introduction

Psychosocial ill health in society is growing rapidly. The transition to parenthood is a joyful event for the majority of new mothers and fathers; however, it can include depressive symptoms and parental stress (1–3), which can negatively affect themselves, their partner, and not least their child (4–6). Depressive symptoms and stress in parents are associated with an increased risk of separation (7), which makes it even more important to focus on depressive symptoms and parental stress.

The prevalence of depressive symptoms in early parenthood is reported to be 5%–20% in mothers (5,7,8) and 3%–10% in fathers (6,9) and is associated with problems in children, such as stress and internalizing problems in both boys and girls (10). There is a link between depressive symptoms and parental stress (3,11), and they influence one another over time (12). Mothers often report higher parental stress than fathers (10,12–16); parents with poor health have higher stress levels than parents with good health (17). Predictors of parental stress are: low education, not living with the partner in early pregnancy (16), child’s behaviour problem, low self-esteem, lack of social support, breast-feeding problems, lack of time with the child, and depression (3).

Parental stress affects parents’ health negatively, especially mothers (17). Parents with lower sense of coherence (SOC) report more parental stress than parents with higher SOC (15). A new source of support is websites where parents can get information, advice, and new friends (18). Some parents lack social support and role models (19). Even though governments in many countries support gender equality, studies in early parenthood often focus on mothers. Further, studies considering mothers and fathers in the same family are still rare. The aim of the present study was to determine whether there is an association between depressive symptoms and parental stress among mothers and fathers in early parenthood. Our hypothesis was that parents with depressive symptoms had higher parental stress compared with parents without depressive symptoms.

## Materials and methods

**Figure 1.**
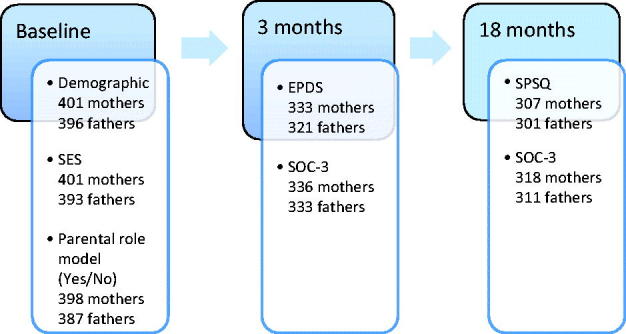
Participants and instruments used from baseline to 18 months after childbirth. SES = socio-economic status; EPDS = Edinburgh Postnatal Depressive Scale; SOC = Sense of Coherence; SPSQ = Swedish Parenthood Stress Questionnaire.

The BiT study (*Barnhälsovård i Tiden*, Child Health Care Today) was a longitudinal cohort study in the county of Västmanland, Sweden, which has a population of about 250,000. The participants were Swedish-speaking parents of children born in the years 2004–2006 from eight Child Health Centres CHCs. During this period, 521 children were born in the study region. The CHC nurses gave all Swedish-speaking new parents oral and written information about the study and asked if they would like to participate. The parents were assured that their participation was voluntary and that they could withdraw from the study at any time, with no effects on their child’s health care. The Central Ethics Committee in Stockholm approved the study. A baseline questionnaire was distributed to the parents by the nurse during their first visit to the CHC and was then returned by the parents in prepaid envelopes. After 3 weeks, a reminder was sent by post, and a second reminder was given by telephone after 5 weeks. Three and 18 months after their children’s births, new questionnaires and return envelopes were sent to the parents’ homes, with reminders as at baseline. The parents were informed that it was important to complete the questionnaires individually. The parents were recruited consecutively, regardless of whether or not the child was their first. Figure 1 provides an overview of the participants and instruments using a timeline from baseline to 18 months after childbirth. These parents have been used for a previous study, but now other extended measures have been carried out (20).

### Baseline questionnaire

The baseline questionnaire contained demographic questions about the parent’s age, the child’s sex, whether or not it was the first child, and the parent’s education level and socio-economic status (SES) (divided into three categories: manual workers, non-manual employees, and self-employed). The parents were also asked whether they had a parental role model with the question ‘Have you had any role model for your role as a mother/father?’; the response options were: ‘Yes’ or ‘No, I have no role model’. This was followed by ‘If yes, who?’

### Three-month questionnaire

*Edinburgh Postnatal Depressive Scale (EPDS).* The EPDS is a screening instrument to identify depressive symptoms (21), validated among mothers and fathers (22) and translated into several languages including Swedish. The scale includes 10 items with a total score of 0–30, where a higher score indicates more depressive symptoms. To estimate a person’s level of depressive symptoms, all 10 questions must be answered. A cut-off point of ≥10 is recommended to identify risk of postnatal depression. In the statistical analyses, EPDS scores were treated as continuous data, and a cut-off point of ≥10 was used.

*Sense of Coherence (SOC).* To measure SOC, we used the three-question instrument SOC-3 suggested by Lundberg and Nyström Peck (23). The three questions address: Manageability (‘Do you usually see a solution to problems that others find hopeless?); Meaningfulness (‘Do you usually feel that your daily life is a source of personal satisfaction?)’; and Comprehensibility (‘Do you usually feel that things that happen in your daily life are hard to understand?’). The response alternatives were ‘No’ (2 points), ‘Yes, sometimes’ (1 point), or ‘Yes, usually’ (0 points); with reversed scoring for comprehensibility. The total score is 0–6, where a score of ≤2 indicating a strong SOC and ≥3 indicating a poor SOC (24).

### Eighteen-month questionnaire

*Swedish Parenthood Stress Questionnaire (SPSQ).* The SPSQ measures parental stress and consists of 34 items within five sub-areas. *Incompetence* focuses on general experiences of care-giving, feelings of incompetence in the parental role, and the difficulty of parenthood. *Role restriction* is about interests and activities restricted by parental responsibilities. *Social isolation* includes social contacts outside the family. The sub-area *Spouse relationship problems* describes the social experiences within the family. *Health problems* deals with the parents’ physical health such as physical fitness, fatigue, and infections. The response options range from ‘Strongly disagree’ to ‘Strongly agree’ on a scale from 1 to 5, with a total possible score of 170 (25). Higher scores indicate higher stress.

### Statistical analyses

Descriptive statistics for the variables are presented as a percentage (number) except for age, which is presented as mean and standard deviation (SD). Comparisons between mothers and fathers were analysed using statistical methods for paired data, because in the context data from the mother and the father who are a couple are dependent. The association between EPDS scores and SPSQ scores in mothers and fathers was assessed using the Spearman’s rank correlation (*ρ*), and the occurrence of depressive symptoms and SPSQ scores was assessed with the Wilcoxon signed-rank test. Linear regression analysis was performed to calculate the regression coefficients (*β*) with corresponding 95% confidence intervals for the outcome SPSQ scores of the mothers and fathers. We first performed univariable analyses of all relevant independent variables. Variables with *P* < 0.2 were then considered in the multivariable analyses. These were performed with forward and backward stepwise selection (5% for inclusion and 5.1% for exclusion) to protect against potential collinearity that could disturb the analyses. Adjusted *R*^2^ was used to identify the proportion of the total variability explained by the model. A residual goodness-of-fit test was used to test the overall fit of the logistic regression model. SPSS (version 20) was used for the statistical analyses, and statistical significance was set at *P* < 0.05 (two-sided).

## Results

The participants’ demographic data, SES, parental role model status, and SOC are presented in Table 1.

**Table 1.** Parents’ age, whether this was their first child, education, socio-economic status (SES), whether parental role model, and sense of coherence (SOC).

	Mothers	Fathers
Parent’s age, mean (SD) (*n*)	30 (5) (401)	33 (6) (396)
First child, % (*n*)		
Yes	40 (161)	44 (175)
No	60 (240)	56 (221)
Education, % (*n*)		
Comprehensive school, 9 years	6 (26)	6 (25)
High school, ≤12 years	60 (241)	74 (290)
University, >12 years	34 (133)	20 (77)
SES, % (*n*)		
Manual workers	59 (235)	70 (277)
Non-manual workers	38 (151)	22 (86)
Self-employed	4 (15)	8 (30)
Parental role model, % (*n*)		
Yes	53 (210)	71 (276)
No	48 (188)	29 (111)
SOC at 3 months, % (*n*)		
Strong	88 (296)	84 (278)
Poor	12 (40)	16 (55)
SOC at 18 months, % (*n*)		
Strong	86 (274)	84 (259)
Poor	14 (44)	17 (52)

### Depressive symptoms 3 months after childbirth

The EPDS score was significantly higher for the mothers (mean 5.5, SD 4.3) than for the fathers (mean 4.0, SD 3.8) (*P* < 0.001). Overall, 18% (*n* = 59) of the mothers and 9% (*n* = 28) of the fathers had an EPDS score ≥10. The results indicate that nearly a quarter (23%) of the children had at least one parent with depressive symptoms (not shown in the table). The correlation between the mothers’ and fathers’ EPDS scores was *ρ* = 0.296 (*P* < 0.001).

### Parental stress 18 months after childbirth

The mothers estimated higher total parental stress than the fathers (*P* < 0.001) (Table 2). Both mothers and fathers had their highest level of stress in the sub-area ‘Role restriction’. The mothers had the lowest level of stress in the sub-area ‘Social isolation’, and the fathers in the sub-area ‘Incompetence’. The mothers perceived higher levels of stress than the fathers did in all sub-areas except for ‘Social isolation’, where the fathers perceived higher stress. The correlation between the mothers’ and fathers’ SPSQ scores was *ρ* = 0.387 (*P* < 0.001).

**Table 2.** Swedish Parenthood Stress Questionnaire: five sub-area scores in mothers and fathers 18 months after childbirth.

Sub-area	Number of mothers/fathers	Mothers (mean ± SD)	Fathers (mean ± SD)	*P* value^a^
Incompetence	314/311	2.1 ± 0.6	1.9 ± 0.6	<0.001
Role restriction	312/308	3.3 ± 0.8	3.1 ± 0.8	<0.001
Social isolation	316/310	2.0 ± 0.6	2.2 ± 0.6	<0.001
Spouse relationship problems	314/310	2.2 ± 0.9	2.1 ± 0.7	0.004
Health problems	316/311	2.6 ± 0.8	2.5 ± 0.7	0.027
Total^b^	307/301	2.4 ± 0.5	2.3 ± 0.5	<0.001

a Wilcoxon signed-rank test.

b Mothers and fathers who answered all sub-areas of parental stress.

### Depressive symptoms 3 months and parental stress 18 months after childbirth

The correlation between the mother’s EPDS score and SPSQ score was *ρ* = 0.467 (*P* < 0.001), and between the mother’s EPDS score and father’s SPSQ score it was *ρ* = 0.312 (*P* < 0.001). The correlation between the father’s EPDS score and mother’s SPSQ score was *ρ* = 0.170 (*P* = 0.005), and between the father’s EPDS score and father’s SPSQ score it was *ρ* = 0.489 (*P* < 0.001) (not shown in the table).

### Linear regression analyses

Significant predictors in the multivariable regression analyses of mother’s SPSQ score at 18 months were mother’s EPDS score at 3 months, mother’s SOC at 18 months, and father’s SPSQ score at 18 months (Table 3). Significant predictors of father’s SPSQ score at 18 months were father’s EPDS score at 3 months, father’s SOC at 18 months, and mother’s SPSQ score at 18 months (Table 3). The results from the univariable regression analyses are presented in Supplementary Tables I and II (available online).

**Table 3.** Multivariable regression analysis for mothers’ and fathers’ Swedish Parenthood Stress Questionnaire (SPSQ) scores 18 months after childbirth.

	SPSQ score		
	*β* (95% CI)	*t* statistic	*P* value
Mothers (*n* = 264)^a^			
Mother’s EPDS score at 3 months	0.04 (0.02–0.05)	5.75	<0.001
Mother’s SOC at 18 months	0.52 (0.37–0.67)	6.70	<0.001
Father’s SPSQ score at 18 months	0.21 (0.11–0.32)	3.98	<0.001
Fathers (*n* = 252)^b^			
Father’s EPDS score at 3 months	0.05 (0.04–0.07)	6.98	<0.001
Father’s SOC at 18 months	0.20 (0.06–0.39)	2.87	0.004
Mother’s SPSQ score at 18 months	0.29 (0.19–0.36)	5.05	<0.001

a Adjusted *R*^2^ 35.9%.

b Adjusted *R*^2^ 30.9%.

## Discussion

The main finding for both mothers and fathers was the association between the parents’ depressive symptoms and parental stress. The mother’s parental stress after 18 months was positively associated with her own depressive symptoms and/or SOC and/or the partner’s parental stress. This was similar for the fathers. As is well known, Sweden is one of the most gender-equal countries in the world in terms of extensive and egalitarian parental leave policies (26). Although many studies confirm differences between mothers and fathers during early parenthood, we found no significant difference between genders concerning parental stress in the present study. Own depressive symptoms and poor SOC affect parental stress for both mothers and fathers. This is in accordance with Edhborg et al., who showed that Swedish fathers with depressive symptoms experience the first year after childbirth similarly to mothers, with turbulent everyday life involving parental stress and lack of social support (13). Furthermore, we state that a partner’s stress affects the other parent’s stress. We speculate that a parent’s stress can affect the partner’s stress because in Sweden both parents share the duties in the family more than previously, as found by Strandh and Nordenmark (27). Perhaps a feeling of losing control could occur if a parent who was used to taking decisions alone is now required to involve the partner. Dissatisfaction with partner support and parental stress are stronger predictors of poor health in fathers than in mothers (17), which may partly explain why the partner’s stress affects the other parent’s stress as described in our results.

The association we found between depressive symptoms and parental stress is in accordance with Saisto et al. (3). A possible explanation may be that a person who experiences depressive symptoms might be more susceptible to parental stress. We also found that parents with poor SOC experienced higher parental stress than did parents with strong SOC. A possible explanation may be that some new parents do not find their situation manageable, with the child taking a lot of their time, and therefore experience more stress. The highest level of parental stress was in the sub-area of ‘Role restriction’ for both mothers and fathers, which is in accordance with other studies (11,15). Parents with poor SOC have a strained economic situation and come from lower social classes (28), which can also contribute to parental stress.

Social support (contact with friends and relatives) has a positive effect on SOC (28). Therefore, maintaining contact with friends and relatives could be important for new parents. Today, parents seek friends through social media and various websites, but this seems to create more parental stress, especially for mothers (18). Perhaps they require more practical support than advice about parenthood. This is in accordance with Saisto et al. (3), who describe parents’ lack of support in early pregnancy. In future studies, it could be interesting to investigate how parental stress and child-care are affected by social media, which might put pressure on parents as they attempt to live up to all the demands of society and their friends.

The mothers experienced more depressive symptoms and higher parental stress than the fathers, which is consistent with previous studies (15,29). The mothers perceived higher total parental stress than the fathers in all sub-areas except for ‘Social isolation’, where the fathers perceived higher parental stress than mothers did. A possible explanation may be that mothers experience greater conflicts between work and household demands than fathers (27). Even though Sweden has one of the most comprehensive and egalitarian parental leave policies in the world (26), mothers still use three-quarters of the parental leave taken (30). Another possible explanation for women experiencing more parental stress may be that they can express their feelings better than men, as they are more used to expressing emotions.

A Swedish study found that one partner’s depressive symptoms affect the other partner’s bonding with the child (29). In the present study, we found that a parent’s parental stress affected the other parent’s stress; however, a parent’s depressive symptoms did not affect the partner’s stress. We must emphasize how important it is that parental stress affects not only the individual but also the partner and ultimately the child’s well-being. The parents’ well-being obviously affects the child’s well-being, and the child’s well-being affects the well-being of the parents, which can lead to a vicious circle that affects family health adversely.

### Strengths and limitations

Studies often focus on mothers’ experiences of parenthood, but studies focusing on the fathers’ experiences are rare. A strength of the present study is that a large number of mothers and fathers of the same child participated. Another strength is that parents from eight different CHCs participated, and this reduced the risk of influences of individual nurses.

The first limitation is that the parents who answered the questionnaires might have fewer depressive symptoms and parental stress than the non-responding parents, as motivation and strength are required to complete the questionnaires. However, two advantages of using questionnaires are that they only take a short time to complete and they have simple scoring systems. A second limitation is that only Swedish-speaking parents were included. Finally, the result might not be generalizable to contexts other than that described in the present study.

## Conclusions

In the present study, we found an association between mothers’ and fathers’ depressive symptoms and parental stress during early parenthood. However, a parent’s stress obviously affects the partner, and this knowledge is important for health professionals as well as parents. This knowledge can be useful for health professionals in preventing and detecting depressive symptoms and parental stress, and for developing interventions. Both mothers and fathers should receive support and guidance during pregnancy and while bringing up their children to optimize the conditions for raising the child.
